# Reduced incidence of arrest following an extreme risk protection order among respondents in California

**DOI:** 10.1093/pnasnexus/pgag184

**Published:** 2026-05-23

**Authors:** Veronica A Pear, Julia P Schleimer, Aaron B Shev, Garen J Wintemute, Shannon Frattaroli, April M Zeoli

**Affiliations:** Centers for Violence Prevention, Department of Emergency Medicine, University of California Davis School of Medicine, Sacramento, CA 95817, USA; Centers for Violence Prevention, Department of Emergency Medicine, University of California Davis School of Medicine, Sacramento, CA 95817, USA; Center for Firearm Injury Prevention, University of Washington School of Medicine, Seattle, WA 98104, USA; Centers for Violence Prevention, Department of Emergency Medicine, University of California Davis School of Medicine, Sacramento, CA 95817, USA; Centers for Violence Prevention, Department of Emergency Medicine, University of California Davis School of Medicine, Sacramento, CA 95817, USA; Center for Gun Violence Solutions, Johns Hopkins Bloomberg School of Public Health, Baltimore, MD 21202, USA; Department of Health Management and Policy, University of Michigan School of Public Health, Ann Arbor, MI 48109, USA

**Keywords:** extreme risk protection orders, red flag laws, gun violence, arrest, health policy

## Abstract

Extreme risk protection orders (ERPOs) are used to disarm individuals making threats of interpersonal harm, but little is known about respondents’ pre- and postorder criminal behavior. We sought to quantify ERPO respondents’ rate of arrest vis-à-vis timing of the order. This was a retrospective cohort study of Californian respondents with ERPOs issued 2016–2019 and for whom we received ERPO court case files (*n* = 679). Arrest and legal firearm purchasing information was obtained from the California Department of Justice, while contextual information about the respondents and their cases was abstracted from ERPO case files. We used adjusted Poisson models to estimate incident rate ratios (IRRs) for arrest (any arrest and arrest for violence, firearm offenses, and firearm violence) comparing rates 6 months before the ERPO was issued to rates while the ERPO was in effect and 6 months after it expired. Respondents were 14–89 years old, 91.9% were men, and 59.9% were non-Hispanic white. Most cases (70.6%) included interpersonal violence threats. Half of respondents (49.8%) legally purchased a firearm prior to the ERPO and 3.4% purchased firearms in the 6 months following the ERPO's expiration. Respondents were significantly less likely to be arrested while the ERPO was in effect than during the 6 months prior to it being issued (IRRs ranged from 0.20 [firearm violence] to 0.45 [any arrest]). These associations persisted after the ERPO expired. Low incidence rate ratios for arrest after ERPOs were issued suggest ERPOs may prevent arrest for criminal behaviors, including violence perpetration, among respondents.

Significance statement In this retrospective cohort study of individuals issued an extreme risk protection order (ERPO; “respondents”) in California, 2016–2019, incident rates of arrest for any offense, violence, firearm-related offenses, and firearm violence while the order was in effect were significantly lower than rates during the 6 months preceding the order (incident rate ratios ranged from 0.20 for firearm violence to 0.45 for any arrest). Associations persisted after the orders expired. These findings suggest that ERPOs may reduce criminal behavior resulting in arrest, including violence, among respondents in California. States without ERPO laws should consider adopting them.

## Introduction

There is an ongoing epidemic of firearm violence in the United States, resulting in 43,593 deaths in 2024 alone (the most recent year of data available) (1). Extreme risk protection orders (ERPOs), known as gun violence restraining orders in California, are designed to prevent firearm violence by temporarily preventing the purchase and possession of firearms by individuals judged to be at high risk of harming themselves or others. Currently, 24 jurisdictions have adopted an ERPO law. In 2016, California became the third state to adopt the law and was the first to base ERPOs on restraining orders (rather than risk warrants), a model followed by all subsequently adopting states.

ERPOs are intended to prevent firearm violence by temporarily preventing individuals from accessing the most lethal means of self- and other-directed violence during transient periods of heightened risk. However, their effectiveness could be undermined by underuse, poor implementation, or respondents (those subject to the order) accessing firearms through extralegal means. To date, studies quantifying the effectiveness of ERPO laws have primarily focused on firearm suicide. For this outcome, descriptive and analytic findings generally point to protective effects among respondents and at the population level (2–8).

Less is known about outcomes related to interpersonal violence, despite ERPO laws primarily being enacted in response to mass shootings, and nothing is known about respondents’ firearm purchasing behavior. Descriptive studies have found that ERPOs have been used to restrict firearm access from individuals who made threats of multiple victim or mass shootings (8, 9). In addition, a few studies have estimated the population-level effectiveness of ERPO laws at reducing interpersonal violence, with mixed results (4, 10, 11). Respondent outcomes related to arrest (including for violence)—an approximation, albeit imperfect, of violence perpetration—have previously been described for Connecticut, Indiana, and Washington. Compared with the year prior to the ERPO, the proportion of respondents arrested increased while the order was in effect in Washington and during the year after the order was issued in Indiana (6, 12); it stayed the same during the year after issuance in Connecticut (5). It is unknown whether these findings hold in other states, but given differences in the law and its implementation, heterogeneity is expected.

The current study sought to fill these gaps by describing respondent firearm purchasing and criminal arrests vis-à-vis the timing of the ERPO, drawing on administrative data from the California Department of Justice (CA DOJ). To quantify changes in the rate of arrest, we compared arrests during the ERPO period and after it expired with arrests before the order was issued. Additionally, we provide contextual information for these outcomes and expand on our prior work describing early ERPO cases (8) using abstracted data from ERPO court case files for respondents with orders issued 2016–2019. Findings will be of interest to violence preventionists, policymakers, and those involved with ERPO implementation.

## Methods

A summary of California's ERPO law is provided in the Supplemental Methods.

### Study design and population

This was a retrospective cohort study of all ERPO respondents issued an ERPO in California between 2016 January 1 and 2019 December 31 for whom we were able to obtain court case records (62% of respondents), which allowed us to precisely determine when each respondent's ERPO was in effect and provide rich context for the outcomes of interest. Arrests were captured from 6 months (180 days) before each respondent's first ERPO to 6 months after the end of their last ERPO in the primary analyses; legal firearm purchasing was described for the pre-ERPO period and up to 1 year after the order expired.

### Data and measures

All ERPO respondents with orders issued between 2016 and 2019 were identified with data from the California Restraining and Protective Order System (CARPOS), maintained by CA DOJ. We used identifiers in CARPOS to request, for all respondents: (i) ERPO case files from courts across the state, (ii) Criminal Offender Record Information (i.e. rap sheets) from CA DOJ, and (iii) mortality and state of residence data from LexisNexis Risk Solutions. To identify the cause of death, we requested death certificates from the state of death for all respondents with a mortality record in LexisNexis. We also obtained respondents’ legal firearm transfers in California by linking them to CA DOJ's Dealer Record of Sale (DROS) data using probabilistic matching with name and date of birth.

Key descriptive measures included respondent and case details abstracted from the court case files (defined in Table S1) and legal firearm transfers among respondents vis-à-vis the timing of the ERPO. Transfers include anything indicating the respondent took ownership of a firearm, including private party transfers, pawn redemptions, dealer sales, and out-of-state registrations. Attempted purchases resulting in a denial after a background check are also recorded. The DROS data are nearly comprehensive for legal firearm transfers (excluding only certain intrafamilial transfers); handgun transactions have been recorded since before the system was digitized in the mid-1970s, and long gun transaction records have been mandatory since 2014. Our DROS data begin in 1996. Unfortunately, transfers for safekeeping—the type that would be generated for transfers related to the ERPO itself—are not recorded in DROS.

The primary exposure was time relative to receiving an ERPO, measured as (i) the 6 months prior to the first order being issued, (ii) the time between when the first order was issued and the last order ended, during which an ERPO was active, and (iii) the 6 months following the end of the last order (due to termination or expiration). The exposure excludes the precipitating event window, defined as the date the first order was issued ±4 days, following the published literature (6, 12). Some respondents with multiple ERPOs had gaps between orders; the person-time and outcomes corresponding to these gaps contributed to ERPO-period time in the primary analysis and to post-ERPO time in a sensitivity analysis.

The primary outcomes were arrest for any offense, violence, any firearm offense, and firearm violence. As in previous studies, we defined offenses as violent following the World Health Organization's definition of violence, which includes offenses considered violent by the Federal Bureau of Investigation (13, 14). Firearm involvement was determined via firearm-specific offenses, firearm modifiers, and mention of firearms in comments. Firearm offenses included firearm violence and nonviolent firearm crimes.

We manually abstracted the ERPO court case files to characterize respondents, case circumstances, and ERPO process details, as has previously been described (15). In a second round of coding, we additionally abstracted dates that ERPOs were in effect for each respondent, using data in the court case files, supplemented by information in CARPOS; dates were abstracted by two coders, and disagreements were settled through group discussion. Contextual case information and outcome data were summarized with descriptive statistics.

### Statistical analyses

Multivariable analyses of arrest vis-à-vis timing of the ERPO used Bayesian Poisson regression models controlling for respondent age at ERPO issuance, race, and gender. Race was included to account for systematic factors that result in minoritized individuals being at higher risk for arrest than white individuals; due to small cells when disaggregating by race, we could only coarsely measure race as white vs. another race. Models included a random intercept for each respondent and a person-month offset. Arrest rates while an ERPO was in effect and up to 6 months after the last ERPO expired were compared with rates in the 6 months leading up to the first ERPO. Individuals were censored upon death or moving out of state (identified with LexisNexis data). We examined the 6 months pre- and post-ERPO to better account for the acuity of the crisis leading to the order and conducted a sensitivity analysis examining a 1-year pre- and post-ERPO period.

Models were run separately for each of our four arrest outcomes (any arrest, violence, firearm-related, and firearm violence). We also explored potential heterogeneity in these associations by stratifying by the directionality of the threat (self-directed, other-directed, or both) at the precipitating event and whether there was documentation of firearms being removed pursuant to the ERPO. A sensitivity analysis explored heterogeneity by race and ethnicity, which was limited by smaller counts for some groups; we therefore stratified results for Black, Hispanic, White, and all other respondents.

We conducted two additional sensitivity analyses. The first examined the primary associations of interest using the full cohort of ERPO respondents, 2016–2019. These analyses relied on CARPOS data since we did not have access to court records for all cases; CARPOS only provides the order issue date, so end dates were inferred based on the standard length of the order type each respondent received (21 days for emergency and temporary orders and 1 year for final orders, see Supplemental Methods). These dates are therefore less accurate than those used in the primary analysis. The second analysis was limited to respondents who were issued a final ERPO, which was intended to remove the influence of individuals with very high rates of arrest resulting from short person-time contributions.

This study was approved by the Institutional Review Board at the University of California, Davis.

## Results

Censoring events were relatively rare in this cohort: one person died in the precipitating event window, six died and four moved out of state during the ERPO period, and two moved out of state within 6 months of the ERPO ending.

### Demographics

Table 1 presents respondent demographic and risk characteristics. There were 679 respondents to ERPOs in California (with 683 cases), 2016–2019, for whom we were able to obtain court case records. They had a mean age of 40.7 years (SD: 15.8 years), ranging from 14 to 89 years old at the time of their first order. A large majority (91.9%) of respondents were men. More than half (59.9%) were White, 19.0% were Hispanic, and 10.3% were Black. Most respondents (*n* = 627) were first issued an ERPO in 2018 or 2019 (Fig. S1) and a plurality were from San Diego County (*n* = 321; Fig. S2).

**Table 1:** California ERPO respondent characteristics, 2016–2019.

	Respondents (*n* = 679) *n* (%)
Age^a^	
Mean (SD)	40.7 (15.8)
Range	14–89
Under 18	18 (2.7)
Gender	
Man	624 (91.9)
Woman	52 (7.7)
Other	1 (0.1)
Unknown	2 (0.3)
Race/ethnicity^b^	
Asian	36 (5.3)
Black	70 (10.3)
Hispanic/Latinx	129 (19.0)
Native American/Alaskan Native	2 (0.3)
Native Hawaiian/Pacific Islander	3 (0.4)
White	407 (59.9)
Another race	21 (3.1)
Unknown	17 (2.5)
Military veteran	
Yes	64 (9.4)
Cognitive condition^b^	
Specific mental health diagnosis	113 (16.6)
Vague statement about mental illness	173 (25.5)
Irrational or erratic behaviors	143 (21.1)
Cognitive decline from traumatic brain injury	4 (0.6)
Age-related cognitive decline	9 (1.3)
Impaired from a medical illness other than dementia or Alzheimer's	4 (0.6)
Impaired cognitive abilities from an unstated cause	2 (0.3)

^a^Age at first order; four people had two separate ERPOs.

^b^Not mutually exclusive.

### Case details

Table 2 presents details of the precipitating event. Over half (56.8%) of cases were in response to threats of interpersonal violence alone, while 12.6% were for threats of self-harm alone; another 13.8% included threats of both self- and other-directed violence. In the remaining cases, the target of the threat was unclear. Fourteen percent of cases (*n* = 97) included multiple victim/mass shooting threats. Among those who made interpersonal violence threats, about a quarter (28.6%) were directed at intimate partners. About one-in-three respondents unlawfully or recklessly used, displayed, or brandished a firearm at the precipitating event, and 76.3% possessed a firearm.

**Table 2:** Precipitating event and ERPO process details, 2016–2019.

	Cases (*n* = 683) *n* (%)
Target of harm	
Self only	86 (12.6)
Others only	388 (56.8)
Self and others	94 (13.8)
Unclear/neither	115 (16.8)
Target of other-directed harm^a^	Among those who threatened or used violence against others (*n* = 482)
Intimate partner	138 (28.6)
Family member (not intimate partner)	61 (12.7)
Minor	28 (5.8)
Law enforcement	40 (8.3)
Hallucination	15 (3.1)
Other target	243 (50.4)
Unclear	79 (16.4)
Threatened multiple victim/mass shooting	
Yes	97 (14.2)
Unlawful or reckless use, display, or brandishing of a firearm	
Yes	212 (31.0)
Substance use	
Alcohol	93 (13.6)
Drugs (other than alcohol)	47 (6.9)
Harm to animals	
Yes	4 (0.6)
Irrational/erratic behavior	
Yes	117 (17.1)
Firearm access	
Possessed firearms at time of precipitating event	521 (76.3)
Had access but no possession	21 (3.1)
Dispossessed before the precipitating event	19 (2.8)
Recently gave their firearms to someone	11 (1.6)
No firearm access	35 (5.1)
Possession unknown	91 (13.3)
Petitioner type	
Law enforcement	664 (97.8)
Immediate family (nonintimate partner)	6 (0.9)
Intimate partner^b^	6 (0.9)
Other	1 (0.1)
Unknown	6 (0.9)
Respondent harmed someone during firearm removal	
Yes	1 (0.1)
Firearms removed pursuant to ERPO	
Yes	341 (50.0)
Additional actions at precipitating event	
State mental health proceedings (5150)	155 (22.7)
Arrest	106 (15.5)
Final order hearing	
Yes	498 (71.6)
No	26 (3.8)
Unknown	159 (23.3)
Outcome of final order hearing	Among those with a hearing (*n* = 498)
Granted/stipulated	389 (78.1)
Denied	18 (3.6)
Dismissed	86 (17.3)
Other	3 (0.6)
Unknown	2 (0.4)
Legal representation at hearing	Among those with a hearing (*n* = 498)
Petitioner	376 (75.5)
Respondent	79 (15.9)

^a^Not mutually exclusive.

^b^Includes spouses, dating partners, and people with a shared child.

Table 2 also presents ERPO process details. The great majority of cases—97.8%—were petitioned for by law enforcement. Case files indicated that firearms were removed pursuant to the ERPO in 50.0% of cases. Other actions taken in conjunction with the ERPO service included state mental health proceedings (22.7%) and arrest (15.5%).

### Firearms

Table 3 presents details of ERPO respondents’ legal firearm purchases, measured with DROS. Three hundred thirty-seven (49.8%) respondents had legally acquired a total of 1,383 firearms in California at any time prior to their first ERPO (mean [SD] firearms per purchaser: 4.1 [7.5]). Ten percent of respondents acquired firearms in the 6 months preceding the order, and only a small proportion of respondents purchased firearms after the order expired (3.4% by 6 months and 4.6% by 1 year). No firearms were purchased, nor were they attempted to be purchased, while the ERPOs were in effect. However, one respondent purchased multiple firearms between one ERPO expiring and another being issued.

**Table 3:** California ERPO respondent legal firearm transfers,^a,b^ 1996–2020.

	Respondents^c^
All legal firearms pre-ERPO	(*n* = 679)
Any transfers, *n* respondents (%)	338 (49.8%)
Legal firearms obtained, *n* firearms	1,383
Firearms obtained per respondent, mean (SD)	2.0 (5.7)
Firearms obtained per purchaser, mean (SD)	4.1 (7.5)
Legal firearm transfers within 6 months pre-ERPO	(*n* = 679)
Any transfers, *n* respondents (%)	68 (10.0%)
Legal firearms obtained, *n* firearms	111
Firearms obtained per person, mean (SD)	0.2 (0.6)
Firearms obtained per purchaser, mean (SD)	1.6 (1.1)
Legal firearm transfers within 6 months post-ERPO^d^	(*n* = 668)
Any transfers, *n* respondents (%)	23 (3.4%)
Legal firearms obtained, *n* firearms	66
Firearms obtained per person, mean (SD)	0.1 (0.7)
Firearms obtained per purchaser, mean (SD)	2.9 (2.8)
Legal firearm transfers within 1 year post-ERPO^d^	(*n* = 668)
Any transfers, *n* respondents (%)	31 (4.6%)
Legal firearms obtained, *n* firearms	87
Firearms obtained per person, mean (SD)	0.1 (0.8)
Firearms obtained per purchaser, mean (SD)	2.8 (2.5)

^a^Long gun transfers were not recorded until 2014.

^b^Transfers include private party transfers, pawn redemptions, dealer sales, and out-of-state registrations.

^c^Denominators consist of all respondents who contributed any person-time during the period of interest.

^d^One respondent purchased several firearms after one ERPO expired and before another was issued. They are included as post-ERPO transfers.

### Arrests

Table S2 presents descriptive data on arrests among ERPO respondents vis-à-vis timing of the ERPO. The number of respondents arrested ranged from 158 (23.3%) for any arrest in the 6-month pre-ERPO period to two (0.3%) for firearm violence arrest in the 6-month post-ERPO period. The proportion arrested for any cause, violence, firearms, and firearm violence were always higher in the pre-ERPO window compared with the precipitating event, ERPO, or post-ERPO windows; however, rates of arrest per person-month were substantially higher during the precipitating event window. For all arrest types, the pre-ERPO arrest rate was higher than the rate during and after the ERPO.

Table 4 displays the adjusted incident rate ratios (IRRs) for arrest during the ERPO period and up to 6 months after the ERPO ended compared with the 6 months before the ERPO was issued. Arrest rates for any offense, violence, firearms, and firearm violence were all significantly lower while the ERPO was in effect compared with the 6 months preceding the order, with IRRs ranging from 0.45 (95% CI: 0.36, 0.57) for any arrest to 0.20 (95% CI: 0.11, 0.37) for firearm violence. These associations persisted after the order expired for all arrest categories. Associations were similar when we stratified by directionality of the threat; however, point estimates were further from zero for those making both self- and other-directed threats compared with those making other-directed threats alone. The former group was quite small though and estimates were sometimes less precise. Self-directed threats alone could not be modeled due to convergence issues. Associations were very similar between those with and without documentation of firearm removal in their court case files. Similarly, results by race and ethnicity were overall consistent across groups, if not always significant (due to reduced power; Table S3). Notably, similar relative rates between racial and ethnic groups are not obscuring large absolute differences in baseline arrest (the mean monthly pre-ERPO rate of arrest for any cause was 0.051 for White and 0.055 for Black respondents); large differences, which we do not see, could have suggested inequities in ERPO implementation even with similar relative rates between groups.

**Table 4:** Adjusted incidence rate ratio of respondent arrest during and 6 months after the ERPO compared with 6 months before the ERPO.^a^

Offense category	IRR (95% CI)	
	ERPO period vs. pre-ERPO period	Post-ERPO period vs. pre-ERPO period
Arrests		
Any	0.45 (0.36, 0.57)	0.37 (0.28, 0.48)
Violence	0.29 (0.21, 0.40)	0.25 (0.17, 0.37)
Firearm	0.24 (0.16, 0.35)	0.30 (0.19, 0.46)
Firearm violence	0.20 (0.11, 0.37)	0.37 (0.20, 0.65)
Nature of threat^b^		
Other-directed alone (*n* = 388)		
Any	0.43 (0.32, 0.58)	0.39 (0.27, 0.55)
Violence	0.29 (0.19, 0.43)	0.20 (0.12, 0.34)
Firearm	0.22 (0.13, 0.37)	0.28 (0.16, 0.49)
Firearm violence	0.16 (0.07, 0.35)	0.28 (0.12, 0.60)
Both self- and other-directed (*n* = 94)		
Any	0.26 (0.13, 0.48)	0.19 (0.07, 0.44)
Violence	0.11 (0.04, 0.30)	0.21 (0.06, 0.58)
Firearm	0.20 (0.06, 0.52)	0.14 (0.03, 0.53)
Firearm violence	0.07 (0.01, 0.37)	0.25 (0.05, 0.90)
Firearms removed		
Yes (*n* = 341)		
Any	0.46 (0.34, 0.62)	0.35 (0.23, 0.52)
Violence	0.30 (0.19, 0.46)	0.23 (0.11, 0.41)
Firearm	0.23 (0.14, 0.38)	0.26 (0.14, 0.45)
Firearm violence	0.21 (0.09, 0.45)	0.35 (0.15, 0.75)
No (*n* = 338)		
Any	0.45 (0.33, 0.62)	0.38 (0.26, 0.55)
Violence	0.29 (0.18, 0.46)	0.28 (0.16, 0.48)
Firearm	0.25 (0.13, 0.44)	0.37 (0.20, 0.68)
Firearm violence	0.20 (0.07, 0.51)	0.40 (0.16, 0.92)

^a^Models are adjusted for age, race, and gender.

^b^There were four arrests in the post-ERPO period for those with self-directed threats alone (*n* = 86), and no models converged for that subpopulation. Additionally, two of the four cases in which a respondent had multiple ERPOs had differences with respect to the nature of the threat and firearm removal. For one respondent, the nature of the threat was self-directed alone in one case and both self- and other-directed in the other; this was coded as both self- and other-directed for the stratified analysis. For the other respondent, the nature of the threat was coded as unclear in one case and other-directed alone in the other; this was coded as other-directed alone for the stratified analysis. For both respondents, one case indicated firearm removal and the other did not; both were coded as having firearms removed for the stratified analysis.

Our sensitivity analyses suggest that the findings are robust to model specifications. We examined adjusted IRRs for arrest from models (i) examining 1-year pre- and post-ERPO periods (Table S4); (ii) reclassifying time and outcomes between multiple orders to the post-ERPO period (Table S5); (iii) using data from all ERPO respondents 2016–2019 (not limited to those for whom we abstracted court case files; Table S6); and (iv) limited to respondents issued a final ERPO (Table S7). Compared with the primary analyses, point estimates were sometimes attenuated but still significant across models, with overall results substantively similar to the primary analyses.

## Discussion

This retrospective cohort study found that arrest rates for any offense, violence, firearms, and firearm violence were substantially lower during the ERPO period and 6 months after the ERPO ended compared with 6 months prior to issuance, suggesting that ERPOs may be dampening criminal behavior resulting in arrest, including interpersonal violence, among respondents.

Adjusting for demographics, we found that, relative to the 6 months before the order was issued, the incidence rate for arrest was 80% (firearm violence) to 55% (any arrest) lower while the ERPO was in effect and 75% (violence) to 63% (any arrest, firearm violence) lower in the 6 months after the order expired. The findings of reduced rates for any arrest and among respondents without documentation of firearm removal suggest that ERPOs may induce a “chilling” effect on the respondents’ criminal activity and/or that the ERPO prompted respondents to address the underlying causes of their criminalized behaviors. However, without a comparison group, we cannot be sure that the decreases in arrest rates are due to the ERPO rather than confounding factors, for example, a natural decline in risk following a crisis. Unfortunately, the published literature offers little empirical guidance on expected arrest patterns before and after similar crises, limiting our ability to benchmark these trajectories. The potential chilling effect of ERPOs on arrest merits further investigation in future studies using appropriate comparison groups.

We found that 23.3% of California ERPO respondents were arrested for any cause in the 6 months preceding the ERPO. Relative to this, the proportion arrested decreased while the ERPO was in effect (9.9%; mean ERPO duration = 310.4 days) and during the 6 months after it expired (5.8%). Studies describing arrest among ERPO respondents in other states found differing patterns in arrests vis-à-vis timing of the order (5, 6, 12). The most comparable study to ours is that of ERPO respondents in Washington, 2016–2020, which, like California, has ERPO laws modeled after domestic violence restraining orders and uses ERPOs mostly for threats of interpersonal violence (16). That study found a slight increase in the proportion arrested while the ERPO was in effect (19.5%) and a decrease in the year after it expired (10.3%) compared with the proportion arrested in the year prior to the order (18.0%) (12). Divergent findings are likely due to differences in use and implementation between states (e.g. a higher proportion of cases include final orders in Washington than California), which should be investigated further.

The proportion of respondents arrested in the 9-day precipitating event window was 9.1% in our study, 18.0% in Washington (12), 17.0% in Connecticut (5), and 5.1% in Indiana (6). The prevalence of arrests around the precipitating event could indicate that law enforcement is considering arrest as a marker of risk when deciding when to petition for an ERPO and/or that arrest is a common response to behaviors that might qualify someone for an ERPO. At the same time, most respondents—all of whom have been adjudicated to be at high risk—were not arrested, which indicates ERPOs are reaching people and addressing risk in ways the criminal legal system is not.

This is the first study to examine legal firearm purchasing among ERPO respondents. Half of respondents legally purchased a firearm in California at any time prior to the ERPO; this is almost certainly an undercount of access to firearms because these data do not include transfers prior to 1996, long gun transfers before 2014, or unlawful or undocumented firearm acquisitions. Indeed, we found that more than three-quarters of respondents were known to possess a firearm at the time of the ERPO according to the court case files. That about 25% of ERPO respondents possessed firearms not recorded in DROS highlights the importance of law enforcement petitioners and judges going beyond these legal firearm transfer records to determine whether a respondent has access to firearms and to ensure all firearms are removed. We also found that 10% of respondents legally purchased a firearm in the 6 months preceding the order, which may be a particularly serious warning sign for people at high risk of violence (17, 18). In the year after the order expired, few (<5%) respondents purchased firearms; additional research should investigate acquisition behaviors following the end of a firearm prohibition.

### Limitations

The information included in ERPO court case files was often in narrative form and therefore not standardized. Individual petitioners determine what to include in the petition, and that determination may differ among people. Some information is frequently missing in case files, such as firearm removal documentation, which could indicate problems with reporting and/or practice. In addition, we could not capture firearm transfers related to the ERPO; alternative data sources should be examined to determine how often respondents retrieve their firearms after the ERPO expires (given the persistent preventive association with arrest after the order ends, many may choose not to). States should also take steps to improve documentation in ERPO case files (19), which would help practitioners and researchers better understand how ERPOs are being implemented and to what effect.

Additionally, arrest is an imperfect and potentially biased measure of criminalized behavior (20), including interpersonal violence. Racial disparities in police surveillance, contact, and arrest are well documented (21–23); these structural inequities shape who becomes visible to law enforcement and how criminal-legal authority is exercised. While our data do not suggest that Black ERPO respondents were disproportionately arrested compared with their white counterparts in California, it is imperative that future research drawing on different populations also assess whether ERPO-related arrest patterns reflect or reproduce racial inequities to ensure just implementation across jurisdictions and over time. Furthermore, our data are for respondents issued orders 2016–2019, with outcomes extending up to 1 year after the order ended; given the many sociopolitical and policy changes that have taken place in recent years, results may differ with more recent data. Finally, our arrest analysis was designed to measure associations; future studies should evaluate the effectiveness of ERPOs at preventing interpersonal violence using causal methods and appropriate comparison groups.

## Conclusions

This study of ERPO respondents in California, 2016–2019, found that ERPOs were associated with decreased arrest among respondents for any cause, violence, firearm-related offenses, and firearm violence while the ERPO was in effect and in the 6 months after it expired, compared with arrests in the 6 months preceding the order. This study, in conjunctions with the extant literature on ERPOs’ effectiveness at preventing suicide (2, 4, 7), suggests these laws have a meaningful role to play in preventing firearm violence and other criminal behaviors.

## Supplementary Material

pgag184_Supplementary_Data

## Data Availability

Data are not publicly available but can be requested from the California Department of Justice. See https://oag.ca.gov/research-services/request-process for more information.
