# Supplementary material for: Reduced incidence of arrest following an extreme risk protection order among respondents in California
Source: PNAS Nexus. 2026 May 23;5(6):pgag184. doi: 10.1093/pnasnexus/pgag184 (PMC13238727; doi:10.1093/pnasnexus/pgag184)

Supplemental Materials for:

Reduced Incidence of Arrest Following an Extreme Risk Protection Order Among Respondents in California

Veronica A. Pear, Julia P. Schleimer, Aaron B. Shev, Garen J. Wintemute, Shannon Frattaroli, April M. Zeoli

Supplemental Methods

California's ERPO Law

California's ERPO law during the study period, 2016-2019, permitted law enforcement, family, and household members to petition a judge for an order. California has two short-term orders, both of which last for 21 days; temporary orders are available to all petitioners during normal court hours and emergency orders are available only to law enforcement at any time. After 21 days, a hearing is held for the final order; if granted, the order remains in place for 1 year. (In September 2020, the law changed to extend the duration of the final order and allow additional types of petitioners.) If the threat is ongoing at the end of the EPRO period, the petitioner can request a renewal; if granted, the order is extended for another year. While the ERPO is in place, the respondent cannot legally possess or purchase firearms or ammunition.

Table S1. Variables Derived from ERPO Court Case Files

Table Variable	Abstraction Codebook Variables
Age	Age of respondent<br>o Date of birth (MM/DD/YYYY)<br>o Age in years
Gender	Respondent gender<br>o Male<br>o Female<br>o Other_____________<br>o Unknown
Race/Ethnicity	Race/ethnicity of respondent. (Click all that apply)<br>o White<br>o Black or African American<br>o American Indian or Alaskan Native<br>o Asian<br>o Native Hawaiian or Pacific Islander<br>o Latino/Latina/Latinx or Hispanic<br>o Other _________<br>o Unknown

Military Veteran	Does the file indicate that the respondent is currently or was formerly in the armed services (military)?<br>o Yes<br>o No
Mental Illness/Cognitive Decline/Neurodiverse/ Erratic or Irrational Behavior	Does the case file include an indication that the respondent has a mental illness, recurring mental health issue, is neurodiverse, in cognitive decline, or engaged in irrational/erratic behaviors?<br>o Yes<br>o No
Target of Harm	• Were suicide ideations, threats, plans, aborted attempts, or attempts reported to be part of the precipitating event? (click all that apply)<br>o Suicide IDEATIONS were part of the precipitating event<br>o Suicide THREATS were part of the precipitating event<br>o Suicide PLANS were part of the precipitating event<br>o ABORTED suicide attempts were part of the precipitating event<br>o Suicide ATTEMPTS were part of the precipitating event<br>o No ideations, threats, or attempts were part of the precipitating event<br>• What self-harm (not suicide attempt) weapon was used as part of the precipitating event? (click all that apply)<br>o Firearm<br>o Bodily weapon<br>o Other weapon ___________<br>o Unknown<br>o No self-harm was part of the precipitating event<br>• If the USE of violence was reported to have occurred in the PRECIPITATING event, who was the apparent target of that use of violence? (click all that apply)<br>o Intimate partner<br>o Minor<br>o Adult family members (excluding intimate partners)<br>o Family members under age 18 (excluding intimate partners)<br>o Family members of unknown age (excluding intimate partners)<br>o Law enforcement<br>o Other targets________________<br>o Person or group is unclear<br>o Hallucination<br>o Use of violence was not part of the precipitating event

	• If the THREAT of violence was reported to have occurred in the PRECIPITATING event, who was the apparent target of that threat of violence? (click all that apply)<br>o Intimate partner<br>o Minor<br>o Adult family members (excluding intimate partners)<br>o Family members under age 18 (excluding intimate partners)<br>o Family members of unknown age (excluding intimate partners)<br>o Law enforcement<br>o Other targets________________<br>o Person or group is unclear<br>o Hallucination<br>o Threats of violence were not part of the precipitating event
Target of Other-Directed Harm	• If the USE of violence was reported to have occurred in the PRECIPITATING event, who was the apparent target of that use of violence? (click all that apply)<br>o Intimate partner<br>o Minor<br>o Adult family members (excluding intimate partners)<br>o Family members under age 18 (excluding intimate partners)<br>o Family members of unknown age (excluding intimate partners)<br>o Law enforcement<br>o Other targets________________<br>o Person or group is unclear<br>o Hallucination<br>o Use of violence was not part of the precipitating event<br>• If the THREAT of violence was reported to have occurred in the PRECIPITATING event, who was the apparent target of that threat of violence? (click all that apply)<br>o Intimate partner<br>o Minor<br>o Adult family members (excluding intimate partners)<br>o Family members under age 18 (excluding intimate partners)<br>o Family members of unknown age (excluding intimate partners)<br>o Law enforcement<br>o Other targets________________<br>o Person or group is unclear

	o Hallucination<br>o Threats of violence were not part of the precipitating event
Threatened Mass Shooting	Was the reported mass shooting threat part of the PRECIPITATING event?<br>o Yes<br>o No<br>o Not reported
Unlawful or Reckless Use, Display, or Brandishing of a Firearm	What deadly weapon, if any, was unlawfully or recklessly used, displayed, or brandished as part of the PRECIPITATING event, according to the case file? (click all that apply)<br>o Firearm<br>o Other weapon<br>o Unknown weapon<br>o No deadly weapon was unlawfully or recklessly used in the precipitating event
Substance Use	Which substances/alcohol use does the file mention were used by the respondent during the PRECIPITATING event, if any? (click all that apply)<br>o Illicit drugs (except heroin). Does not include use or misuse of prescription drugs or marijuana.<br>o Opioid use (including heroin). Includes any prescription opioids and/or heroin<br>o Marijuana<br>o Vague reference to drugs<br>o Other substance misuse_________________<br>o Alcohol<br>o No substance/alcohol use mentioned during the precipitating event
Harm to Animals	Did the respondent harm animals as part of the PRECIPITATING event?<br>o Yes<br>o No<br>o Unknown
Irrational/Erratic Behavior	Were irrational/erratic behaviors part of the PRECIPITATING event, according to the case file?<br>o Yes<br>o No<br>o Unclear

Firearm Access	According to the case file, did the respondent possess a firearm at the time the petition was filed? (click all that apply)<br>o There is evidence in the file of firearm possession<br>o Respondent was recently dispossessed as part of the ERPO petition process<br>o Respondent was dispossessed by law enforcement before the precipitating event<br>o Respondent recently gave their firearms to someone else<br>o Respondent does not possess or have access to a firearm<br>o Respondent has access to, but does not possess, a firearm<br>o It is unknown whether the respondent possessed a firearm<br><br>Note: Possessed firearms at the time of the precipitating event = "There is evidence in the file of firearm possession" OR "Respondent was recently disposed as part of the ERPO petition process"
Petitioner Type	Type of petitioner<br>o Law enforcement<br>o Immediate family (non-intimate partner)<br>o Intimate partner (spouse, dating partner, shared child)<br>o Health professional<br>o Other _____________<br>o Unknown
Respondent Harmed Someone During Firearm Removal	Is there information in the file indicating the respondent threatened or harmed anyone during the process of removing or relinquishing firearms after an ERPO was granted?<br>o Yes, please describe_______________<br>o No
Firearms Removed Pursuant to ERPO	• Is there information in the file indicating that firearms were removed or relinquished after an ERPO was granted?<br>o Yes<br>o No<br>• According to the case file, did the respondent possess a firearm at the time the petition was filed? (click all that apply)<br>o There is evidence in the file of firearm possession<br>o Respondent was recently dispossessed as part of the ERPO petition process<br>o Respondent was dispossessed by law enforcement before the precipitating event<br>o Respondent recently gave their firearms to someone else<br>o Respondent does not possess or have access to a firearm<br>o Respondent has access to, but does not possess, a firearm<br>o It is unknown whether the respondent possessed a firearm

	Note: Firearms removed pursuant to an ERPO = "Respondent was recently dispossessed as part of the ERPO petition process"
Additional Actions at Precipitating Event	• Were the state proceedings regarding mental health concerns part of the PRECIPITATING event, as reported in the case file.<br>o Yes<br>o No<br>o Unclear<br>• Is there evidence in the case file that the respondent was arrested during the PRECIPITATING event?<br>o Yes<br>o No
Final Order Hearing	Was there a final order hearing? (this includes cases in which there was a stipulation to the final order)<br>o Yes<br>o No<br>o No, petition was dismissed before the final order hearing<br>o Unknown
Outcome of the Final Order Hearing	What was the outcome of the final order hearing?<br>o Granted<br>o Granted – the respondent stipulated to the final order<br>o Denied<br>o Dismissed<br>o Unknown<br>o Other
Legal Representation at Hearing	• Was the respondent represented by an attorney at the final hearing?<br>o Respondent was represented by an attorney<br>o Respondent was not represented by an attorney<br>o Unknown<br>• Was the petitioner represented by an attorney at the final hearing?<br>o Petitioner was represented by an attorney<br>o Petitioner was not represented by an attorney<br>o Unknown

Table S2. Arrests Among ERPO Respondents in California

Arrest Type and Timing[a]	N Respondents Arrested	N Respondents[b]	% Arrested	N Arrests	Total Person-Months	Mean Arrests per Person per Month (SD)
Any						
Pre-ERPO	158	679	23.3%	204	4020.4	0.051 (0.106)
Precipitating Event	62	679	9.1%	73	178.5	0.409 (1.378)
ERPO	67	678	9.9%	150	6939.1	0.025 (0.140)
Post-ERPO	39	668	5.8%	59	4020.4	0.015 (0.071)
Violence						
Pre-ERPO	100	679	14.7%	119	4020.4	0.030 (0.076)
Precipitating Event	45	679	6.6%	52	178.5	0.291 (1.149)
ERPO	32	678	4.7%	47	6939.1	0.012 (0.108)
Post-ERPO	13	668	1.9%	16	3947.6	0.004 (0.030)
Firearm						
Pre-ERPO	82	679	12.1%	96	4020.4	0.024 (0.068)
Precipitating Event	32	679	4.7%	36	178.5	0.201 (0.947)
ERPO	23	678	3.4%	27	6939.1	0.004 (0.032)
Post-ERPO	13	668	1.9%	14	3947.6	0.004 (0.026)
Firearm Violence						
Pre-ERPO	40	679	5.9%	46	4020.4	0.011 (0.048)
Precipitating Event	20	679	2.9%	22	178.5	0.123 (0.734)
ERPO	3	678	0.4%	3	6939.1	0.000 (0.005)
Post-ERPO	2	668	0.3%	2	3947.6	0.001 (0.009)

a. Pre-ERPO includes the 6 months before ERPO until the precipitating event. The precipitating event includes the first ERPO issue date +/- 4 days. The ERPO period includes time while ERPO is in effect. Post-ERPO includes the 6 months after the order expired.
b. Respondents are included in the count if they contributed any person-time to that period. One person died during the precipitating event window (of non-firearm suicide) and 6 died during the ERPO period (2 of non-firearm suicide, 1 of the self-inflicted gunshot wound that precipitated the ERPO, and 3 of other causes). Additionally, 4 moved out of state during the ERPO period and 2 moved out of state in the 6-month post-ERPO period.

Table S3. Sensitivity Analysis: Adjusted Incidence Rate Ratio of Respondent Arrest During and 6 Months After the ERPO Compared with 6 Months Before the ERPO, Stratified by Race[a]

**Offense Category**	**IRR (95% CI)**	
**Race**	**ERPO Period**	**Post-ERPO Period**
Black (N=70)		
Any	0.32 (0.14, 0.71)	0.31 (0.13, 0.71)
Violence	0.16 (0.04, 0.52)	0.14 (0.03, 0.54)
Firearm	0.14 (0.03, 0.56)	0.21 (0.04, 0.80)
Firearm Violence	0.17 (0.02, 1.05)	0.23 (0.02, 1.48)
Hispanic (N=129)		
Any	0.44 (0.27, 0.71)	0.32 (0.17, 0.57)
Violence	0.26 (0.13, 0.51)	0.14 (0.05, 0.35)
Firearm	0.20 (0.08, 0.45)	0.20 (0.07, 0.50)
Firearm Violence	0.06 (0.01, 0.32)	0.10 (0.01, 0.52)
White (N=407)		
Any	0.49 (0.37, 0.65)	0.42 (0.30, 0.57)
Violence	0.38 (0.25, 0.56)	0.38 (0.23, 0.61)
Firearm	0.29 (0.18, 0.47)	0.38 (0.23, 0.63)
Firearm Violence	0.34 (0.16, 0.69)	0.64 (0.31, 1.23)
Other/Unknown (N=73)		
Any	1.04 (0.26, 4.60)	0.26 (0.02, 1.67)
Violence	0.24 (0.02, 2.28)	0.08 (0.00, 1.25)
Firearm	0.11 (0.00, 2.50)	0.59 (0.05, 5.78)
Firearm Violence	0.09 (0.00, 2.49)	0.12 (0.00, 2.41)

a. Models are adjusted for age and gender.

Table S4. Sensitivity Analysis: Adjusted Incidence Rate Ratio of Respondent Arrest During and 1 Year After the ERPO Compared with 1 Year Before the ERPO[a]

**Offense Category**	**IRR (95% CI)**	
**Arrests**	**ERPO Period**	**Post-ERPO Period**
Any	0.81 (0.66, 1.00)	0.50 (0.39, 0.62)
Violence	0.56 (0.41, 0.77)	0.36 (0.26, 0.50)
Firearm	0.46 (0.31, 0.67)	0.37 (0.25, 0.54)
Firearm Violence	0.40 (0.21, 0.71)	0.44 (0.25, 0.74)

a. Models are adjusted for age, race, and gender.

Table S5. Sensitivity Analysis: Adjusted Incidence Rate Ratio of Respondent Arrest During and 6 Months After the ERPO Compared with 6 Months Before the ERPO, Time Between 2+ ERPOs Counted as Post-ERPO Time[a]

**Offense Category**	**IRR (95% CI)**	
**Arrests**	**ERPO Period**	**Post-ERPO Period**
Any	0.70 (0.57, 0.85)	0.36 (0.27, 0.46)
Violence	0.62 (0.47, 0.82)	0.25 (0.16, 0.37)
Firearm	0.50 (0.36, 0.69)	0.29 (0.19, 0.44)
Firearm Violence	0.61 (0.38, 0.98)	0.36 (0.19, 0.63)

a. Models are adjusted for age, race, and gender. Counts of events and person-time were aggregated for each person by the period type: pre-, during-, or post-ERPO period.

Table S6. Sensitivity Analysis: Adjusted Incidence Rate Ratio of Respondent Arrest During and 6 Months After the ERPO Compared with 6 Months Before the ERPO, All Respondents 2016-2019[a]

**Offense Category**	**IRR (95% CI)**	
**Arrests**	**ERPO Period**	**Post-ERPO Period**
Any	0.43 (0.35, 0.54)	0.36 (0.28, 0.47)
Violence	0.28 (0.20, 0.39)	0.24 (0.16, 0.36)
Firearm	0.25 (0.17, 0.37)	0.26 (0.16, 0.39)
Firearm Violence	0.19 (0.10, 0.35)	0.34 (0.19, 0.60)

a. Models are adjusted for age, race, and gender.

Table S7. Sensitivity Analysis: Adjusted Incidence Rate Ratio of Respondent Arrest During and 6 Months After the ERPO Compared with 6 Months Before the ERPO Among Respondents with Final Orders[a]

**Offense Category**	**IRR (95% CI)**	
**Arrests**	**ERPO Period**	**Post-ERPO Period**
Any	0.57 (0.44, 0.72)	0.33 (0.22, 0.47)
Violence	0.41 (0.28, 0.58)	0.25 (0.14, 0.42)
Firearm	0.34 (0.22, 0.53)	0.28 (0.15, 0.50)
Firearm Violence	0.33 (0.18, 0.60)	0.33 (0.14, 0.68)

a. Models are adjusted for age, race, and gender.

Figure S1. Number of New ERPO Respondents by Year and Quarter

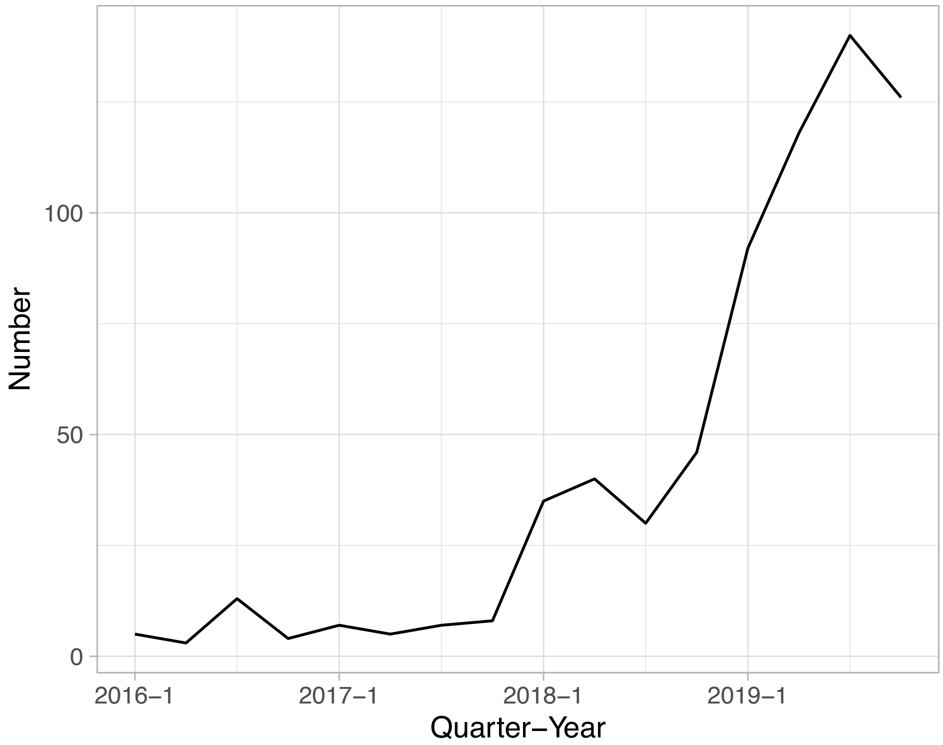

Figure S2. Map of ERPO Respondents by County

A) Counts

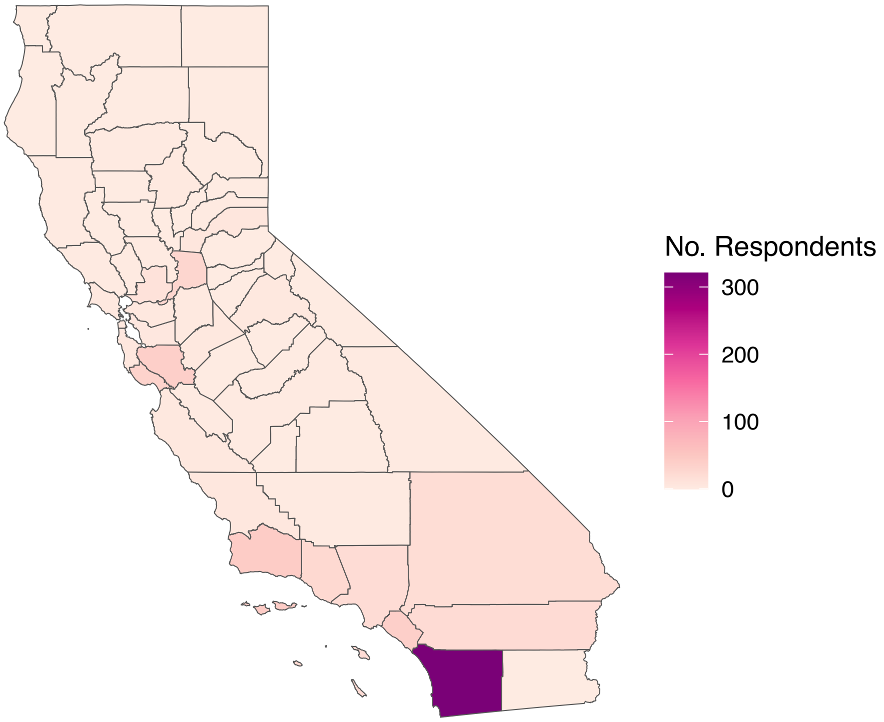

B) Rates

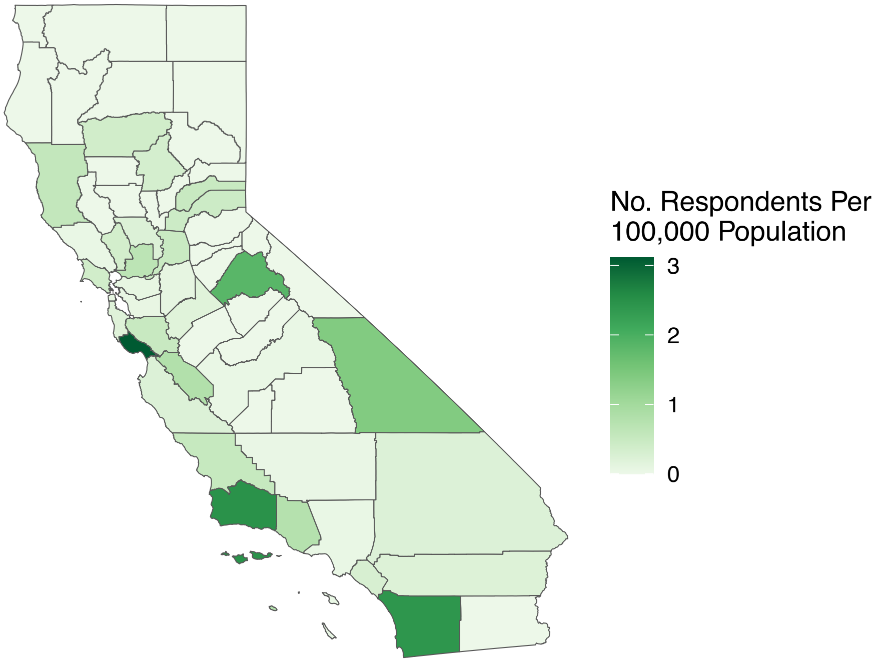

Supplement: pgag184_Supplementary_Data [file pgag184_supplementary_data.pdf]
